# Integrating Social Determinants of Health in Machine Learning–Driven Decision Support for Diabetes Case Management: Protocol for a Sequential Mixed Methods Study

**DOI:** 10.2196/56049

**Published:** 2024-09-25

**Authors:** Seung-Yup Lee, Leslie W Hayes, Bunyamin Ozaydin, Steven Howard, Alison M Garretson, Heather M Bradley, Andrew M Land, Erin W DeLaney, Amy O Pritchett, Amanda L Furr, Ashleigh Allgood, Matthew C Wyatt, Allyson G Hall, Jane C Banaszak-Holl

**Affiliations:** 1 School of Health Professions University of Alabama at Birmingham Birmingham, AL United States; 2 Department of Quality and Patient Safety University of Alabama at Birmingham Medicine Birmingham, AL United States; 3 Department of Care Transitions University of Alabama at Birmingham Medicine Birmingham, AL United States; 4 Cooper Green Mercy Health Service Authority Birmingham, AL United States; 5 Primary Care Line University of Alabama at Birmingham Medicine Birmingham, AL United States; 6 Heersink School of Medicine University of Alabama at Birmingham Birmingham, AL United States; 7 Cardiovascular Institute University of Alabama at Birmingham Medicine Birmingham, AL United States; 8 Informatics Institute University of Alabama at Birmingham Birmingham, AL United States

**Keywords:** diabetes, case management, case manager, social work, case mix, social determinants of health, clinical decision support, decision support, predictive analytics, disparities, health disparities, data warehouse, tertiary care, health care system, chronic disease management

## Abstract

**Background:**

The use of both clinical factors and social determinants of health (SDoH) in referral decision-making for case management may improve optimal use of resources and reduce outcome disparities among patients with diabetes.

**Objective:**

This study proposes the development of a data-driven decision-support system incorporating interactions between clinical factors and SDoH into an algorithm for prioritizing who receives case management services. The paper presents a design for prediction validation and preimplementation assessment that uses a mixed methods approach to guide the implementation of the system.

**Methods:**

Our study setting is a large, tertiary care academic medical center in the Deep South of the United States, where SDoH contribute to disparities in diabetes-specific hospitalizations and emergency department (ED) visits. This project will develop an interpretable artificial intelligence model for a population with diabetes using SDoH and clinical data to identify which posthospitalization cases have a higher likelihood of subsequent ED use. The electronic health record data collected for the study include demographics, SDoH, comorbidities, hospitalization-related factors, laboratory test results, and medication use to predict posthospitalization ED visits. Subsequently, a mixed methods approach will be used to validate prediction outcomes and develop an implementation strategy from insights into patient outcomes from case managers, clinicians, and quality and patient safety experts.

**Results:**

As of December 2023, we had abstracted data on 174,871 inpatient encounters between January 2018 and September 2023, involving 89,355 unique inpatients meeting inclusion criteria. Both clinical and SDoH data items were included for these patient encounters. In total, 85% of the inpatient visits (N=148,640) will be used for training (learning from the data) and the remaining 26,231 inpatient visits will be used for mixed-methods validation (testing).

**Conclusions:**

By integrating a critical suite of SDoH with clinical data related to diabetes, the proposed data-driven risk stratification model can enable individualized risk estimation and inform health professionals (eg, case managers) about the risk of patients’ upcoming ED use. The prediction outcome could potentially automate case management referrals, helping to better prioritize services. By taking a mixed methods approach, we aim to align the model with the hospital’s specific quality and patient safety considerations for the quality of patient care and the optimization of case management resource allocation.

**International Registered Report Identifier (IRRID):**

DERR1-10.2196/56049

## Introduction

Diabetes is a major source of morbidity and mortality in the United States. According to the Center for Disease Control and Prevention, over 38 million people or 11.6% of the US population have diabetes [1-3]. Poorly managed diabetes leads to poor glycemic control and associated significant complications that require hospitalization. Furthermore, having diabetes can complicate recovery from other medical and surgical morbidities leading to a greater likelihood for longer hospitalization, repeat hospitalizations, and emergency department (ED) use [4-6]. Since 2010, hospitalizations associated with diabetes and related conditions have increased likely due to an increase in prevalence and deterioration in diabetes control [7,8].

There is convincing evidence that social determinants of health (SDoH), or the conditions where people are born, grow, live, work, and age are associated with the prevalence of diabetes as well as outcomes among those with a diagnosis [9-11]. SDoH frameworks suggest that there are multiple causal pathways that link SDoH to outcomes. Some frameworks appropriately acknowledge upstream regional determinants such as the political environment or government policy having impact on downstream determinants such as living conditions and health behavior which in turn impact health outcomes [12]. Another framework uses educational attainment as a starting point leading to various other determinants such as health literacy, work, income, social supports, and social standing, which in turn are related to health behaviors, access to nutrition, and health care, which are then linked to health outcomes [13].

Strategies for moderating the negative impacts of diabetes involve participation in lifestyle changes related to diet and exercise, the use of drugs, as well as screenings for acute and chronic complications [14]. The effectiveness of these strategies is impacted by SDoH as well as underlying other comorbidities. Therefore, health systems focused on reducing diabetes-related ED and inpatient use are working to incorporate the social context within programs aimed at helping patients with diabetes improve their health outcomes [11].

However, there are 3 important caveats to consider in applying an SDoH framework in attempting to explain inequalities in diabetes health outcomes. First, although descriptions and pictures of these frameworks imply that the relationship between SDoH factors and health are linear, in truth the interactions between these variables are complicated and difficult to isolate. Second, SDoH factors are both at the individual (eg, a person’s wage) and societal level variables (eg, community infrastructure). Third, case managers and nurses must figure out how to assess patients’ SDoH characteristics and health needs to properly identify patients who will most benefit from case management and then design a program specifically tailored to that patient’s needs [15,16]. There are constraints in a health system’s ability to provide comprehensive case management care [17,18] as exemplified at the University of Alabama at Birmingham Health System (UABHS), where the limited availability of registered nurse (RN) case managers for identifying and managing posthospitalization high-risk diabetes cases hinders effective case management. These 3 factors combined make it difficult to identify and address the individual and community level factors that might have a nuanced relationship on desired patient outcomes.

Decision support tools and predictive analytics hold promise for assisting health systems in identifying populations most at risk and developing targeted interventions. These tools can take a holistic approach [19-21], incorporating both health status and SDoH and address the nuanced relationships between these variables (eg, nonlinearity and interactions) and their combined effect on health outcomes. However, although predictive analytic and decision support systems can easily identify high-risk diabetes cases [22,23], many of these tools only relied on health system data overlooking the nuances of daily practice and operations and user perception, hindering the implementation of a developed tool. Stakeholders, including health care professionals, struggle to support implementation due to a lack of clear insights into the performance and clinical and operational relevance of these systems.

Our study responds to these significant research gaps and practical challenges by proposing the development of a Proactive Risk Assessment Decision Support (PRADS) model through a mixed-methods (qualitative and quantitative) approach. The quantitative component will use classification performance metrics and their visualization to identify and then validate a risk stratification model for predicting an ED visit post discharge among patients with diabetes. A focus group and survey will be done with nurse manager, case managers, clinicians, and care transition leaders at UABHS to qualitatively assess the model’s fit with clinical and operational workflows and its potential impact on quality of care (motivated by [24,25]). Combined quantitative outcomes and qualitative feedback will be used to refine diabetes case management protocols, making them more effective and aligned with real-world health care delivery.

This new tool will be used to support UABHS’ case managers in identifying and prioritizing patients with diabetes with a higher risk of subsequent ED use post hospitalization. The data-driven predictive model will amalgamate repositories of patient data that include both clinical and SDoH factors [26,27]. This predictive approach has the potential to automate case management referrals in a data-driven manner, helping to better prioritize services and use.

## Methods

**Figure 1:**
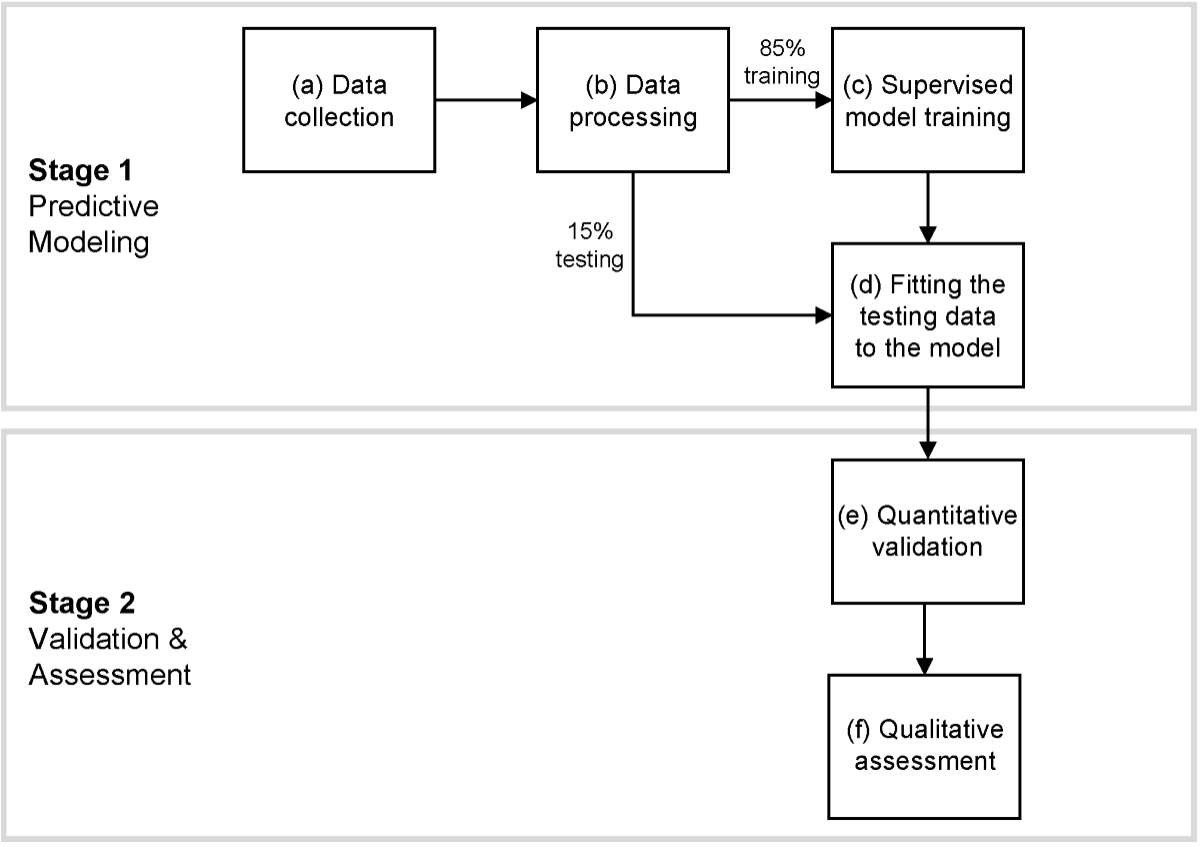
Study framework.

UABHS is a large public hospital with an academic mission and a level 1 trauma center. The health system has implemented a case management program using RN case managers with training in diabetes care to improve outcomes for those recently hospitalized with diabetes. With over 50,000 patients with diabetes receiving care in the health system, the case management referrals prioritize cases based on two highly exclusive, preset clinical rules at point of discharge: (1) hemoglobin A_1c_ (HbA_1c_) > 10.5% or (2) blood glucose > 300 mg/dL and pH < 7.3. Our methodology aims to create an expanded set of data-driven referral criteria and validate the criteria through a mixed methods evaluation. Figure 1 depicts our study framework. The research will be conducted by a large multidisciplinary team, including experts from the 5 groups below:

1. Case management providers, including directors of care transitions and diabetes case management RNs.
2. Primary care physicians from internal and family medicine.
3. Quality and patient safety experts, including the associate chief quality officer and quality outcome nurses.
4. Health data scientists and system analysts.
5. Large-scale electronic health record (EHR) experts, in this case the Director and staff for the University of Alabama at Birmingham (UAB) Enterprise Data Warehouse.

The large-scale EHR experts have already helped develop a process for standardizing data acquisition to use long term in the implementation of case management system changes. Our study framework requires several stages of data collection and analysis as shown in Figure 1 and described below.

### Stage 1: Predictive Modeling

We will model the risk of a posthospitalization ED visit among inpatients with diabetic concerns admitted to UABHS between January 2018 and September 2023 using patient predictors and ED visit history, including health outcome post hospitalization. Patient predictors include both SDoH and clinical factors collected from the UABHS EHR repository. This approach aims to prioritize patients based on their likelihood of visiting the ED within a specific timeframe. Predictions will be retrospectively generated for the daily inpatient mix at UABHS, aligning with the current practices in case management.

### Steps (a and b): Developing Measures for Analysis

Our initial study population includes those who were hospitalized with the following conditions (in Textbox 1) recorded as of October 2023.

**Laboratory test item and ICD-10 (International Statistical Classification of Diseases, Tenth Revision) codes included in the sample inclusion criteria.**

- Hemoglobin A_1c_ level≥6.5% OR
- Diabetes mellitus due to underlying condition (E08) OR
- Drug or chemical induced diabetes mellitus (E09) OR
- Type 1 diabetes mellitus (E10) OR
- Type 2 diabetes mellitus (E11) OR
- Other specified diabetes mellitus (E13) OR
- Elevated blood glucose level (R73) OR
- Hypoglycemia (E16.2) OR
- Foot ulcer (L97.509) OR
- Wound infection (T14) OR
- Gastroparesis (K31.84)

The proposed criteria in Textbox 1 are more inclusive than the current restrictive case management referral criteria that are based exclusively on HbA_1c_, blood glucose, and pH levels; yet, the sample size is not too expansive to perform large-scale machine learning modeling.

**Figure 2:**
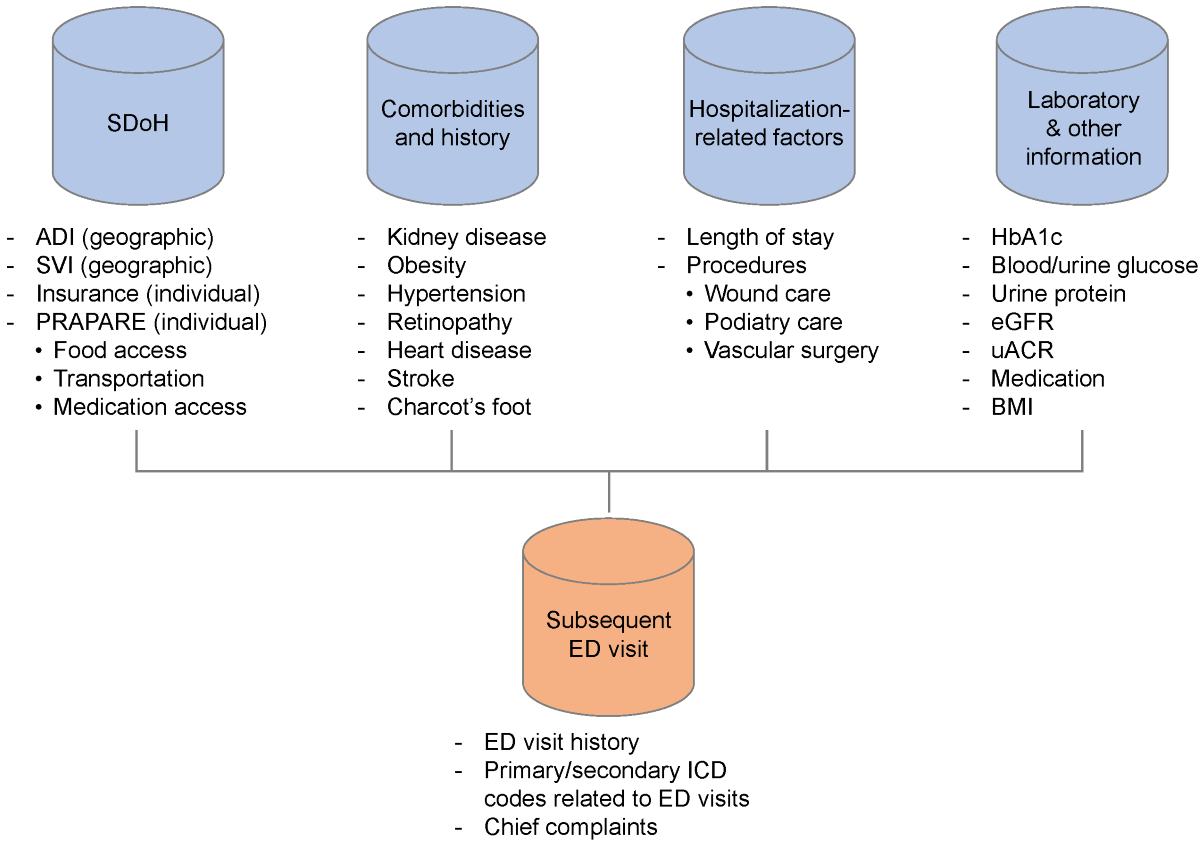
Data items used to build the proactive risk assessment decision support model. ADI: Area Deprivation Index; ED: emergency department; eGFR: estimated glomerular filtration rate; HbA1c: hemoglobin A1c; ICD: International Classification of Diseases; PRAPARE: Protocol for Responding to and Assessing Patients’ Assets, Risks, and Experiences; SVI: Social Vulnerability Index; uACR: urine albumin-creatinine ratio.

Figure 2 lists all the key diabetes-related clinical and socioeconomic factors collected from the UAB Enterprise Data Warehouse for the sample in this study. We choose to include variables that measure both individual levels of risk and societal or area-level measures of risk since SDoH frameworks suggest that both types of variables have some impact on the risk of poor health. Indeed, at our own institution we have recognized that individuals from certain communities are more likely to return to the ED compared to individuals at other communities. What is not clear from the literature is the relative impact of each type of variable on the specific risk of the individual. For example, there is some evidence that area level measures such as the Area Deprivation Index (ADI) may predict individual risk [28-30], and there is also evidence that such area level variables cannot predict individual risk [31]. Further, even if the area-level variables demonstrate less importance in predicting ED use, program planners must understand the context in which an individual lives. Area-level factors (including, the ADI and Social Vulnerability Index [SVI]) were collected from the Neighborhood Atlas and Agency for Toxic Substances and Disease Registry databases. The ADI and SVI were selected to reflect essential community-level barriers and resources that impact diabetes outcomes [32].

The importance of certain kinds of individual level factors (eg, race, insurance status, prior medical history, and comorbidities) has long been established as predictors of risk [33]. However, other kinds of socioeconomic data will help identify specific patient needs. The Protocol for Responding to and Assessing Patients’ Assets, Risks, and Experiences (PRAPARE) [34] is a self-reported survey completed by patients during health care encounters, and include reports of access to housing and transportation, food security, and whether the patient is employed. The significance of these factors in the diabetes context has been discussed in the literature [35-37]. At UABHS, the PRAPARE survey has been implemented in the ED and inpatient settings as part of the health system’s initiative, and our study will use PRAPARE data where available via the EHR repository for the study population. We will also incorporate individual-level variables included in administrative data and the medical record such as insurance status, use and visit history, age, gender, race, and ethnicity. To ensure the robustness of our findings, we will analyze and report on the missingness of the PRAPARE data items. This approach will help us assess potential biases in data collection and address their impact on the study results.

While area-level factors provide a broader view of SDoH for patients, individual-level SDoH factors also provide information of a patient’s unique socioeconomic situation and together should be useful in predicting the most vulnerable and at-risk patients.

Our retrospective predictive modeling approach will use 85% of the data (148,640 inpatient visits) for training (learning from the data) and the rest of the data (26,231 inpatient visits) for validation (testing). Data elements will be cleaned and transformed based on expert clinicians’ input (knowledge-driven) to create categorical and numerical variables for modeling. Categorical variables will be created from comorbidities, medications, and PRAPARE data, while ADI and SVI scales will be treated as numerical. Missing information on categorical variables will be coded as Not Measured. Our outcome variable, whether the individual returned to the ED post discharge for diabetic concerns, is a binary variable that will be taken from either the primary or secondary diagnosis at admission (see Textbox 1).

### Step (c) Supervised Model Training

Given the availability of a wide range of EHR data elements at UABHS covering both predictors and the outcome variable, we choose to conduct supervised learning of patterns between the predictors and outcomes [38]. Our predictive model develops a scale of care urgency (eg, the Emergency Severity Index [39]) that identifies the likelihood that a patient may return to the ED within a specified timeframe post discharge, which can inform prioritization of patient cases in case management. We factor in a set of covariates *X_i_* at the time that the patient (say, patient i) is hospitalized (*T_i_*). These covariates include SDoH and the other 3 types of variables represented in Figure 2.

**Figure 3:**
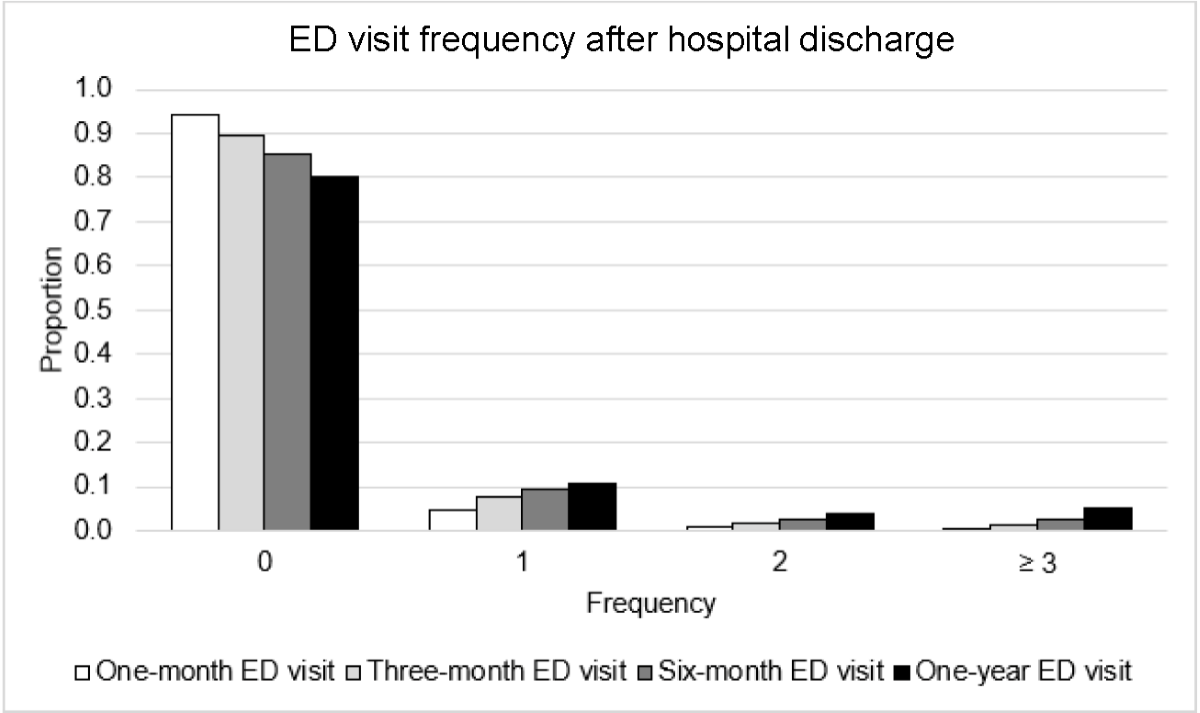
Diabetes-related posthospitalization emergency department (ED) visit frequency.

In our modeling framework, defining *t* is an important task to ensure the practical applicability of prediction outcomes. The grouped bar graph in Figure 3 shows a preliminary analysis of the postdischarge diabetes-related ED visit frequency. The x-axis represents the frequency of ED visits from 1 month to 1 year post discharge. The y-axis shows the proportion of inpatients who returned to the ED with diabetic concerns specified in Textbox 1 as either the primary or secondary diagnosis. The result indicates that 5.7% visits to the ED occurred within a month of discharge and 10.6% within 3 months of discharge. Given the overall average of 42 daily hospitalizations with diabetes-related concerns, on average, 2.4 inpatients were likely to return to the ED within 1 month, and 4.5 were likely to return within 3 months. Discussions with the case management and clinical teams suggest that although a 1-month timeframe provided a more manageable number of cases for patient follow-ups, a 3-month period offered greater flexibility and aligned better with the projected capacity for posthospitalization case management. Therefore, we will examine whether a patient *i* will return to the ED post hospitalization between *T_i_* and *T_i_* + 3 months*.*

Using binomial classification structures, we will develop an Interpretable Artificial Intelligence (IAI) framework from the decision tree algorithm. The core algorithm will use a top-down search through the space of possible branches to create a tree-like decision structure [40]. Decision tree algorithms use a measure of information gained from choosing features that split the data best [40,41]. Decision trees can handle nonlinear relationships between predictors and outcome variables and interactions between predictors. Moreover, they generate inherently interpretable models, allowing for easy visualization and interpretation of the decision-making paths, which facilitate the understanding of how decisions are reached. These features make decision trees particularly suitable for meeting our study objectives, incorporating SDoH, and conducting mixed-methods validation. In our study, to be specific, we will apply the Classification and Regression Trees (CART) algorithm that uses the Gini impurity measure as a criterion for splitting [42]. Using the CART algorithm, the IAI model will be trained in a supervised manner (ie, learning from patterns between *X_i_* and the outcome variable). After the model is trained, we will fit the testing data to the trained model to derive the prediction outcome.

### Step (d) Fitting the Testing Data to the Model

**Figure 4:**
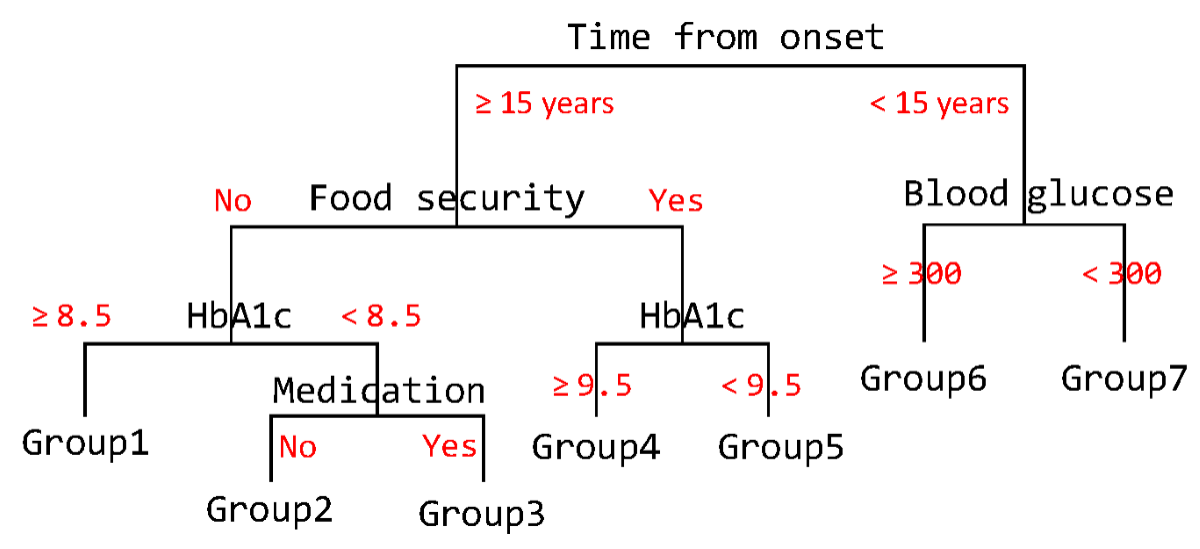
A tree form example. HbA1c: hemoglobin A1c.

A simplified example of trained decision trees is shown in Figure 4. The order of the variables partitioned (branches) and the threshold values that split the branches (in red) are determined in the training process, and the tree structure does not change during the testing step. Depending on the predicted likelihood of an ED admission assigned to a patient during testing, the visit will fall into one of the groups identified by the trained decision tree. For example, the simplified decision tree in Figure 4 shows 7 groups. Each of the groups (in Figure 4, that would be each of the 7 groups) is assigned an estimated probability of experiencing an ED visit as well as Yes/No indicators (for whether the probability leads to ED visit or not).

As represented in Figure 4, our IAI framework enables humans to comprehend the AI’s decisions and facilitates discussion about the prediction outcomes and the variables involved between researchers and practitioners [43]. The prediction outcomes will be the basis for focus group follow-up during Stage 2 of the study.

### Stage 2: Validation of the Model’s Results

**Figure 5:**
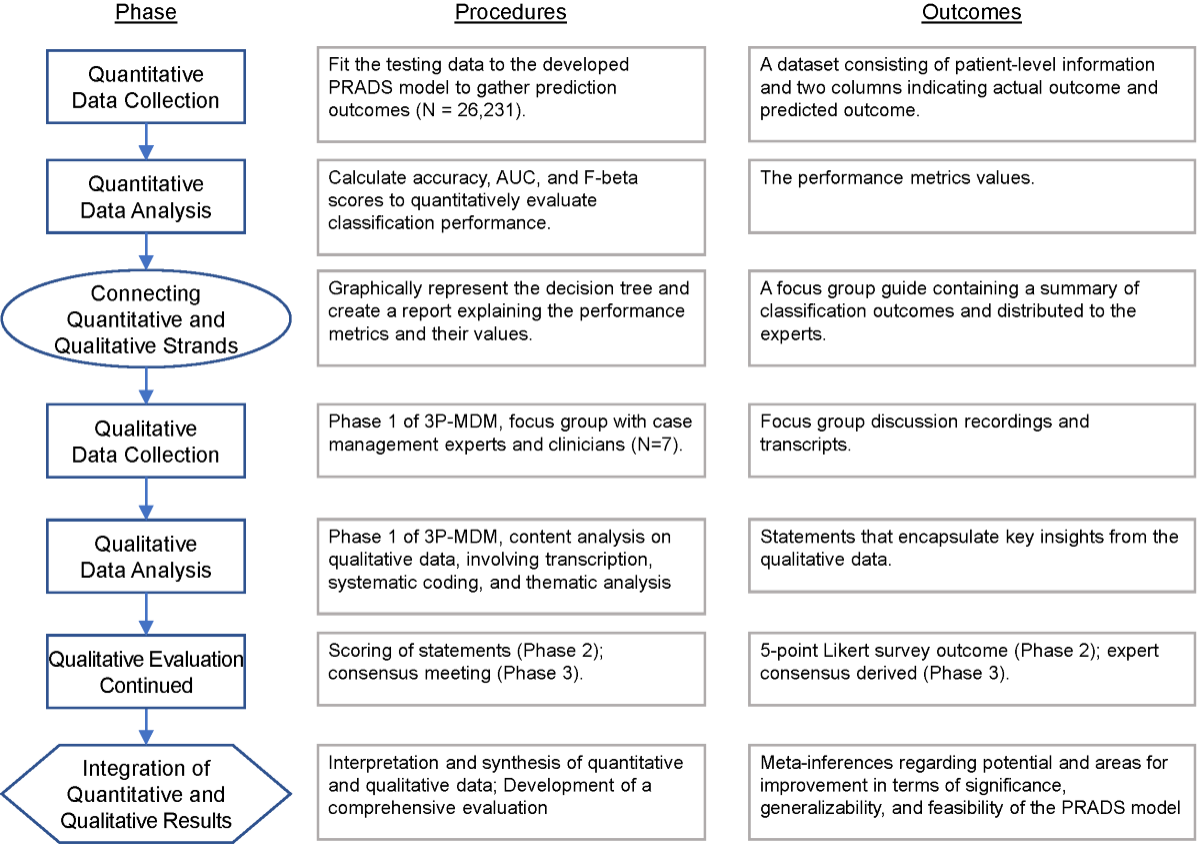
Sequential mixed methods evaluation of the PRADS for Stage 2. 3P-MDM: three-phase modified Delphi method; AUC: are under the curve; PRADS: proactive risk assessment decision support.

The results of our CART analysis will be validated by quantitative performance measures as well as qualitative data from case management, clinician, and quality and patient safety experts. A procedural diagram for our evaluation process is presented in Figure 5. The model development and validation are formative because the evaluation outcomes will be integrated in further development of the PRADS model.

### Step (e) Quantitative Validation of Prediction

For quantitative validation of the PRADS model, predicted outcomes will be generated for the 15% testing data (N=26,231). We will take 3 steps to validate our predicted model. First, prediction accuracy, the most fundamental metric, will be evaluated. Prediction accuracy measures the proportion of true results (both true positives and true negatives) among the total number of cases examined [44]. Prediction accuracy can be misleading, particularly in imbalanced data sets where 1 class (eg, no ED visits within 3 months in our study) significantly outnumbers the other [45]. Second, we will estimate the area under the curve measure, which estimates the area under the receiver operating characteristic curve in a graphical plot illustrating the predictive ability of a binary classifier. Area under the curve is widely used as a measure of validity because it provides an aggregate measure of performance across all binary classification probability thresholds [46]. Lastly, we will estimate the F-beta score, which measures a model’s accuracy both for precision and sensitivity. It can be more informative than prediction accuracy on imbalanced data sets because it considers the model’s ability to identify positive results from the minority class, not just the majority class [47,48]. With beta > 1, the measure puts more emphasis on sensitivity than precision. In our scenario, sensitivity (ie, detection rate) and precision (ie, prediction reliability) deliver their unique implications; therefore, we will evaluate both F-1 and F-2 scores. It will be useful to examine these 3 metrics to provide a well-rounded understanding of the model’s performance.

### Step (f) Qualitative Assessment of Prediction

The qualitative assessment of the model's performance will be a crucial counterpart to our quantitative analysis, ensuring the clinical validity and practical use of our predictions for developing an implementation plan. We will use a 3-phase modified Delphi method (3P-MDM) [49], guided by the Consolidated Framework for Implementation Research (CFIR). The CFIR systemically evaluates factors that can impact the success of implementation in complex settings like health care and is applicable to the early stages prior to implementation [50,51]. Key figures from UAB Medicine Quality and Patient Safety, including the Associate Chief Quality Officer, the Director of Quality Outcomes, and the Quality Outcomes Coordinator, will develop key questions for the initial focus group, which will set the overall direction of our 3P-MDM approach. Details of the 3P-MDM are as follows.

#### Phase 1 of 3P-MDM: Initial Focus Group for Qualitative Feedback

1. Semistructured assessment focus group: The focus group will involve a semistructured discussion session, engaging a panel of experts from the UAB Medicine Department of Care Transition, including the Vice President of Care Transitions, the Senior Director of Care Transition, and an RN case managers, all of whom have extensive experience in case management. Additionally, Family & Community Medicine and Internal Medicine clinicians from UABHS will offer clinical insights from their respective fields. The combined perspectives of these professionals (N=7) will be critical for a comprehensive review of the model’s predictive outcomes, ensuring that the decision-support tool is statistically robust, clinically relevant, and operationally feasible within UABHS. The focus group session will be asked to elaborate on how to implement the model’s predictions based on the five major domains of CFIR:
Intervention characteristics: How do you perceive the strength and quality of the predictive models? Are there specific features that particularly stand out or need improvement to better serve socioeconomically vulnerable groups? Outer setting: What patient needs and resources are being addressed by these models? How does it align with the external policies and incentives in our health care system? Inner setting: How do the models fit into the existing workflow and systems? What is the level of readiness for implementing these models? Characteristics of individuals: From your perspective, how do the models affect your decision-making in patient care? What are the potential barriers and facilitators to using these models? Process of implementation: What strategies would you suggest for the successful implementation of these models in our practice? How can we ensure ongoing adaptations and sustainability?
  Identification of key findings: The discussion will generate rich qualitative data that will be analyzed through content analysis, involving transcription, systematic coding, thematic analysis, and comprehensive reporting. This analysis will highlight key themes and patterns that can offer deep insights into the facilitators and barriers to implementation of the prediction model [52].

#### Phase 2 of 3P-MDM: Structured Evaluation and Ranking

1. Scoring of statements: the derived themes from Phase 1 will be presented back to the 7 experts, who will then score them on a 5-point Likert scale (1 being “strongly disagree” and 5 “strongly agree.” This scale is specifically designed to quantify the importance and applicability of each finding.
2. Ranking of statements: statements will be ranked based on their assigned scores.

#### Phase 3 of 3P-MDM: Expert Consensus and Prioritization

1. Consensus meeting: an expert panel, consisting of 3 experts from case management and 2 primary care physicians, will conduct an anonymous poll to reach a consensus on the most critical findings for model adjustment and implementation. The poll will focus on which ranked findings should be prioritized in further model adjustment and for implementation.
2. Top insights for model adjustment: the identified top statements, as agreed upon by the expert panel, will inform the refinement of the predictive model and guide the implementation process.

This process underscores our commitment to developing a decision-support tool that is not just empirically sound but also pragmatically grounded and clinically endorsed. The qualitative development of an implementation plan, encompassing both the priority rankings and the rich discussions and comments from the focus groups, will provide a comprehensive understanding of the model’s context and importance.

### Ethical Considerations

The University of Alabama at Birmingham Office of the Institutional Review Board for Human Use (UAB Office of IRB) has waived ethics approval. Also, the UAB Office of IRB has waived informed consent to participate. The UAB Office of IRB determined this project is not subject to the Food and Drug Administration’s regulations and is not human participants research. All methods will be carried out in accordance with relevant guidelines and regulations.

## Results

As of December 2023, we have collected data on 174,871 inpatient encounters that occurred from January 2018 to September 2023. These encounters involve 89,355 unique inpatients who have met the criteria in Textbox 1. For this patient population and their respective inpatient encounters, we have fully collected the data items in Figure 2, and data collection was completed as of December 2023.

## Discussion

The anticipated findings of this study would include the predictive capability of the decision tree model, interactions among clinical factors and SDoH, and qualitative assessment results. Our protocol is unique in 3 main aspects. First, the PRADS model will leverage SDoH information to improve diabetes risk stratification. Second, our model results will be interpretable to ensure the users understand how the model reached a recommendation. Third, our model validation and assessment for developing an implementation plan will take a mixed-methods approach, which will be guided by performance metrics (quantitative) and the CFIR (qualitative). Therefore, our protocol will ensure that our findings are grounded in the complex reality of health care delivery, where both numerical data and human insights are equally important. We expect to have the model that is not only statistically sound but also resonates with the needs and constraints of health care providers, ultimately enhancing patient outcomes.

The study has potential limitations. First, its findings, based on one institution, may not be broadly generalizable. Future research could consider comparing different diabetes case management settings, despite challenges due to differences in EHR systems across health care organizations. Second, while our study will include user’s perspectives for model validation, it would still not demonstrate the effectiveness of our approach on patient outcome. This can be done by performing cost-effectiveness analysis on a potential intervention strategy [53], driven by the prediction system or by conducting a pilot implementation of the system to perform prospective data collection and analysis on patient outcome. While analysis on effectiveness is not within the scope of this protocol, future research should address these questions for the successful implementation of the system.

For the development and implementation of the PRADS model within the health system, a comprehensive approach encompassing training, education, system integration, and continuous feedback is critical [54,55]. In our future research, we plan to initiate this with a combination of practical workshops, where case managers engage with a simulated PRADS environment, and theoretical lectures to clarify the model’s computational aspects, especially the use of SDoH data in assessing patient risk. These sessions will allow case managers to voice concerns and discuss practical applications. Simultaneously, we will focus on integrating PRADS into the existing decision-support framework, evaluating its compatibility with current case management strategies, IT infrastructure, and anticipated cost-benefit ratios, for agile development to create a comprehensive system blueprint [56].

As long-term consideration, central to our initiative is the establishment of a robust feedback loop that allows case managers to report discrepancies and contribute insights, keeping the model dynamic and responsive. This will be complemented by thorough documentation, encompassing the model’s design, data processing, and methods of feedback integration, with a strong focus on data sensitivity and patient confidentiality. Ethical considerations will guide our approach, ensuring PRADS augments clinical judgment, and we will develop strategies for enhanced patient engagement in their care planning. Additionally, the PRADS model’s deployment may be implications on health policy, particularly in the context of reimbursement models and value-based care. Continuous research and validation will compare PRADS with traditional risk stratification methods, using longitudinal studies to assess its long-term impact on patient outcomes and health care use.

In summary, the development of the PRADS model will be more than a technological advancement in diabetes care; it can serve as a catalyst for a holistic and nuanced understanding of patient management and a systematic incorporation of human insights into the model development and implementation process. Our model development protocol and the long-term strategy offer a blueprint for enhancing chronic disease management. The modeling and analysis results from this research protocol will be submitted to a medical informatics journal and be discussed in national and international academic conferences. The initial results will serve as preliminary data for follow-up studies, including multi-institutional model development, cost-effectiveness analysis, and prospective data collection and analysis on patient outcome. The research team will pursue extramural funds to fulfill the team’s goal of optimizing chronic disease management and health care resource use.

## Abbreviations

3P-MDM: three-phase modified Delphi method
ADI: Area Deprivation Index
CART: Classification and Regression Trees
CFIR: Consolidated Framework for Implementation Research
ED: emergency department
EHR: electronic health record
HbA1c: hemoglobin A1c
IAI: interpretable artificial intelligence
ICD-10: International Statistical Classification of Diseases, Tenth Revision
PRADS: proactive risk assessment decision support
PRAPARE: Protocol for Responding to and Assessing Patients’ Assets, Risks, and Experiences
RN: registered nurse
SDoH: social determinants of health
UAB: University of Alabama at Birmingham
UABHS: University of Alabama at Birmingham Health System

## Data Availability

The data sets generated and analyzed during the current study are not publicly available because the deidentified electronic health record data are obtained from the UAB Enterprise Data Warehouse and intended to be used for internal purpose only. Data are, however, available from the authors upon reasonable request and with permission of the UAB health system. Dr Seung-Yup (Joshua) Lee and the UAB Enterprise Data Warehouse should be contacted if someone wants to request the data from this study.
